# Mechanisms of pathogenesis induced by bovine leukemia virus as a model for human T-cell leukemia virus

**DOI:** 10.3389/fmicb.2013.00328

**Published:** 2013-11-08

**Authors:** Yoko Aida, Hironobu Murakami, Masahiko Takahashi, Shin-Nosuke Takeshima

**Affiliations:** ^1^Viral Infectious Diseases Unit, RIKENWako, Saitama, Japan; ^2^Laboratory of Animal Health II, Azabu UniversitySagamihara, Kanagawa, Japan; ^3^Division of Virology, Niigata University Graduate School of Medical and Dental SciencesNiigata, Japan

**Keywords:** BLV, HTLV-1, EBL, B-cell lymphoma, Tax, leukemogensis, transactivation, apoptosis

## Abstract

Bovine leukemia virus (BLV) and human T-cell leukemia virus type 1 (HTLV-1) make up a unique retrovirus family. Both viruses induce chronic lymphoproliferative diseases with BLV affecting the B-cell lineage and HTLV-1 affecting the T-cell lineage. The pathologies of BLV- and HTLV-induced infections are notably similar, with an absence of chronic viraemia and a long latency period. These viruses encode at least two regulatory proteins, namely, Tax and Rex, in the pX region located between the *env *gene and the 3′ long terminal repeat. The Tax protein is a key contributor to the oncogenic potential of the virus, and is also the key protein involved in viral replication. However, BLV infection is not sufficient for leukemogenesis, and additional events such as gene mutations must take place. In this review, we first summarize the similarities between the two viruses in terms of genomic organization, virology, and pathology. We then describe the current knowledge of the BLV model, which may also be relevant for the understanding of leukemogenesis caused by HTLV-1. In addition, we address our improved understanding of Tax functions through the newly identified BLV Tax mutants, which have a substitution between amino acids 240 and 265.

## INTRODUCTION

Bovine leukosis was first reported in 1871 as the presence of slightly yellow nodules in the enlarged spleen of cattle (Leisering, 1871). Spleen disruption consecutive to tumor formation is one of the most important clinical manifestations of bovine leukemia. Bovine leukosis is classified into two types, sporadic bovine leukosis (SBL) and enzootic bovine leukosis (EBL), which are characterized by T- and B-cell leukosis, respectively (Gillet et al., 2007). The occurrence of EBL in cattle is much higher than that of SBL (Theilen and Dungworth, 1965; Onuma et al., 1979). Bovine leukemia virus (BLV), which belongs to the *Retroviridae* family and *Deltaretrovirus* genus, is the etiologic agent of EBL, although it remains unknown what causes SBL (Gillet et al., 2007). The natural hosts of BLV are domestic cattle and water buffaloes; however, experimental infection with BLV in sheep can lead to the development of lymphoma (Djilali and Parodi, 1989). Interestingly, BLV is consistently associated with leukemia only in cattle and sheep, even though it can infect many cell lines (Graves and Ferrer, 1976) and can be experimentally transmitted to rabbits (Wyatt et al., 1989; Onuma et al., 1990), rats (Altanerova et al., 1989), chickens (Altanerova et al., 1990), pigs, goats, and sheep (Mammerickx et al., 1981). Most BLV-infected cattle are asymptomatic, but approximately one-third of them suffer from persistent lymphocytosis (PL) characterized by non-malignant polyclonal B-cell expansion and 1–5% of them develop B-cell leukemia/lymphoma after a long latency period (Gillet et al., 2007). On the other hand, sheep that are experimentally inoculated with BLV develop B-cell tumors at a higher frequency and with a shorter latency period than those observed in naturally infected cattle (Ferrer et al., 1978; Burny et al., 1979; Kenyon et al., 1981; Aida et al., 1989). Interestingly, the transformed B-lymphocytes in cattle are CD5^+^ IgM^+^ B-cells (Aida et al., 1993), whereas in sheep they are CD5^-^ IgM^+^ B-cells (Murakami et al., 1994a,b), suggesting that the mechanisms of leukemogenesis induced by BLV may differ (Graves and Ferrer, 1976; Djilali and Parodi, 1989).

BLV is closely related to human T-cell leukemia virus type 1 (HTLV-1), which is the causative agent of adult T-cell leukemia (ATL) and a chronic neurological disorder known as tropical spastic paraparesis or HTLV-1-associated myelopathy HAM/TSP (Gessain et al., 1985; Osame et al., 1986; Gillet et al., 2007). Therefore, studies on BLV may facilitate our understanding of the mechanism of leukemogenesis induced by HTLV-1.

## BLV AND HTLV-1

**FIGURE 1:**
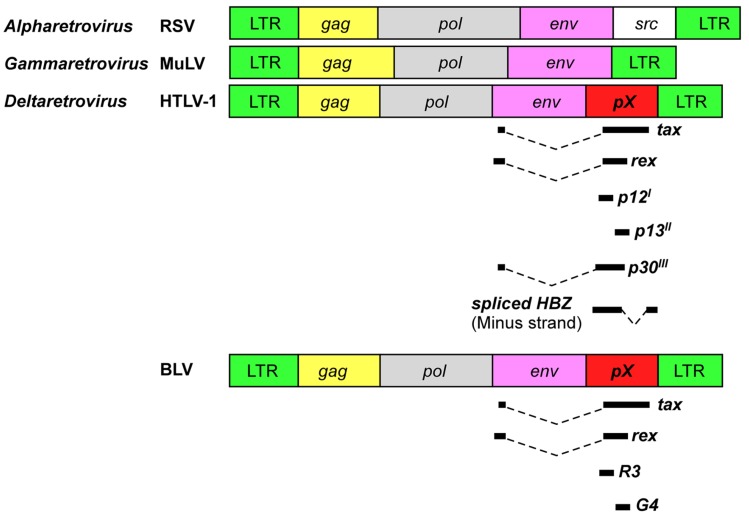
**Schematic representation of genome organization of retroviruses.** HTLV-1 and BLV encode unique regulatory and accessory proteins in the pX region. RSV, Rous sarcoma virus; MuLV, murine leukemia virus.

All retroviruses are encoded by *gag*, *pro*, *pol*, and *env* essential genes, which are necessary for the production of infectious virions, and are flanked by two identical long terminal repeats (LTRs; **Figure 1**). The *gag*, *pro*, *pol*, and *env* genes encode the internal structural proteins of the virion, the viral protease, the reverse transcriptase, and the envelope glycoproteins of the virion, respectively. The genome sequences of BLV and HTLV-1 are different, but have a unique sequence called the pX situated between the *env* gene and the 3′LTR and encoded by the regulatory gene (**Figure 1**). The pX sequence is not of host cell origin; that is, it is not an oncogene. It has been reported that both viruses have an ability to immortalize primary cells *in vitro* (Grassmann et al., 1989; Willems et al., 1990). Because their structure and properties differ from any other class of retroviruses, BLV and HTLV-1 viruses were classified into a new group of retroviruses (Gillet et al., 2007). In both viruses the regulatory proteins Tax and** Rex are encoded in the pX region. The R3 and G4 proteins are encoded in the BLV pX region, while p12^I^, p13^II^, and p30^II^ are encoded in the HTLV-1 pX region (Sagata et al., 1984b; Franchini et al., 2003; **Figure 1**). Interestingly, the HTLV-1 genome codes for HBZ, a unique gene encoded by the minus strand chain (Gaudray et al., 2002; **Figure 1**). The major functions of the viral proteins encoded in the BLV and HTLV-1 pX regions are summarized in **Table 1**. The Tax protein has been extensively studied, and it is believed to play a critical role in leukemogenesis induced by BLV and HTLV-1 (Katoh et al., 1989; Tanaka et al., 1990; Willems et al., 1990). The Rex protein is responsible for nuclear export of viral RNA and promotes cytoplasmic accumulation and translation of viral messenger mRNA in BLV- and HTLV-1-infected cells (Felber et al., 1989). BLV R3 and G4 proteins contribute to the maintenance of high viral load (Willems et al., 1994; Florins et al., 2007). The G4 protein is particularly relevant to leukemogenesis, since it can immortalize primary rat embryo fibroblasts (REFs; Lefebvre et al., 2002). HTLV-1 p12^I^ is similar to the R3 protein, in that it contributes to the maintenance of infectivity (Collins et al., 1998), and both proteins are located in the nucleus and cellular membranes (Gillet et al., 2007). On the other hand, HTLV-1 p13^II^ protein resembles the G4 protein, since both proteins bind to farnesyl pyrophosphate synthetase, which farnesylates Ras (Lefebvre et al., 2002), and the p13^II^ protein promotes Ras-dependent apoptosis (Hiraragi et al., 2005). HTLV-1 p30^II^ protein regulates gene transcription through its interaction with the cAMP responsive element (CRE) binding protein (CREB)/p300 (Zhang et al., 2001). The HBZ protein plays a critical role in the leukemogenesis of HTLV-1, and HBZ knockdown inhibits the proliferation of ATL cells (Satou et al., 2006). However, since the BLV genome does not code for HBZ, it is assumed that the Tax protein plays a central role in the leukemogenesis of BLV.

**Table 1:** Viral proteins are encoded in BLV and HTLV-1 pX regions.

Virus	Viral protein	Major reported functions	Reference
BLV	Tax	Transcriptional activator of viral expression	Derse (1987), Willems etal. (1987), Katoh etal. (1989)
		Oncogenic potential	Willems etal. (1990)
		Activation of NF-kappa B (NF-κB) pathway	Szynal etal. (2003), Klener etal. (2006)
	Rex	Nuclear export of viral mRNAs	Felber etal. (1989)
	G4	The maintenance of high viral load	Willems etal. (1994), Florins etal. (2007)
		Oncogenic potential	Kerkhofs etal. (1998), Lefebvre etal. (2002)
	R3	The maintenance of high viral load	Willems etal. (1994), Florins etal. (2007)
HTLV-1	Tax	Transcriptional activator of viral expression	Kashanchi and Brady (2005)
		Oncogenic potential	Matsuoka and Jeang (2011)
		Induction of DNA damage, cellular senescence and apoptosis	Chlichlia and Khazaie (2010)
		Functional regulation of many cellular proteins by direct binding	Boxus etal. (2008)
	HBZ	Inhibition of HTLV-1 transcription	Lemasson etal. (2007)
		Suppression of the classical pathway of NF-κB	Zhao etal. (2009)
		Enhancement of TGF-β signaling	Zhao etal. (2011)
		Oncogenic potential	Satou etal. (2006), 2011
	Rex	Nuclear export of viral mRNAs	Felber etal. (1989)
	p12^I^	Maintenance of viral infectivity	Collins etal. (1998)
		Activation of nuclear factor of activated T-cells (NFAT) pathway	Ding etal. (2002)
	p13^II^	Suppression of viral replication	Andresen etal. (2011)
		Interaction with farnesyl pyrophosphate synthetase	Lefebvre etal. (2002)
		Activation of Ras-mediated apoptosis	Hiraragi etal. (2005)
	p30^II^	Suppression of viral replication	Nicot etal. (2004)
		Regulation of gene transcription by binding with p300	Zhang etal. (2001)
		Enhancement of Myc transforming potential	Zhang etal. (2001)

The infection route of BLV and HTLV is by horizontal and vertical transmission. BLV is transmitted via direct contact (Kono et al., 1983), milk, and insect bites (Ferrer and Piper, 1978), while HTLV-1 is transmitted via milk and sexual intercourse (Bangham, 2003). Moreover, the artificial transmission of BLV is caused by iatrogenic procedures such as dehorning, ear tattooing, and reuse of needles (Hopkins and DiGiacomo, 1997), whereas the artificial transmission of HTLV-1 is caused by blood transfusion and needle sharing among drug abusers (Robert-Guroff et al., 1986). Since cell contact is required for the efficient transmission of both BLV and HTLV-1, cell-free infection by these viruses is believed to be very inefficient, most probably due to virion instability (Voneche et al., 1992; Johnston et al., 1996; Igakura et al., 2003).

**FIGURE 2:**
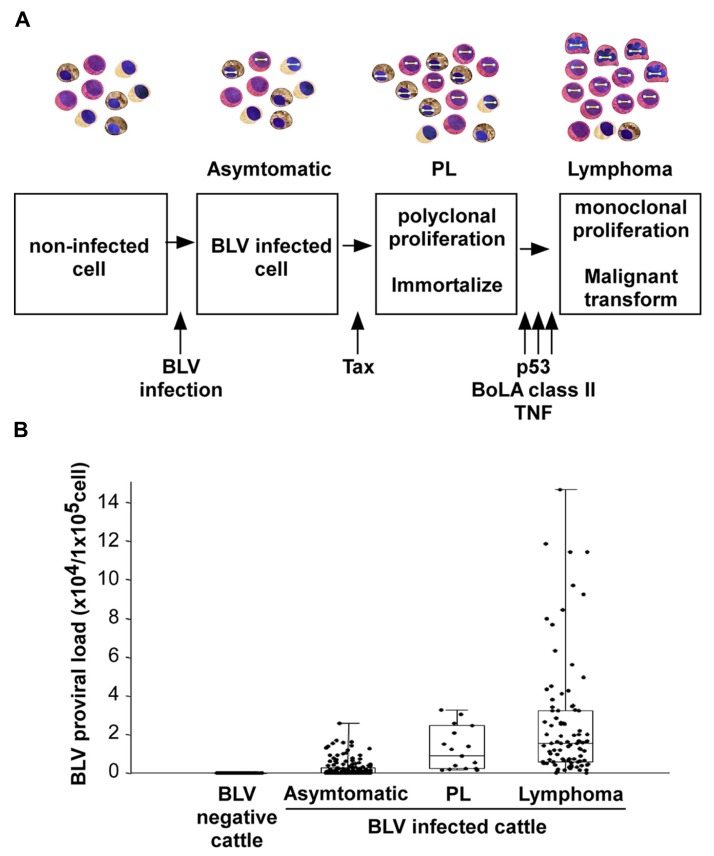
**BLV-induced leukemogenesis is a multistep process.** **(A)** An infection with BLV is characterized by three progressive stages of disease: asymptomatic stage, persistent lymphocytosis (PL), and lymphoma. BLV infects to cells non-specifically. Among them, BLV Tax protein immortalizes a part of BLV-infected cells, probably only CD5^+^ IgM^+^ B-cells, and induces polyclonal proliferation of the cells. However, the Tax protein does not have the ability to transform the cells. For lymphoma to develop, a malignant transformation needs to occur with the help of host factors, such as p53 mutation, TNF-α activities or bovine leukocyte antigen (BoLA) class II phosphorylation. **(B)** The provirus load increases with disease progression.

As shown in **Figure 2**, an infection with BLV is characterized by three progressive stages of disease, including an asymptomatic stage, PL, and lymphoma. Most BLV-infected cattle are asymptomatic, but approximately one-third of them suffer from PL characterized by a permanent and relatively stable increase in the number of B-lymphocytes in the peripheral blood. PL is considered to be a benign form of the disease resulting from the accumulation of untransformed B-lymphocytes. Finally, 1–5% of BLV-infected cattle develop B-lymphoma in various lymph nodes after a long latency period (Schwartz and Levy, 1994; Florins et al., 2008). Although BLV can also infect CD4^+^ T-cells, CD8^+^ T-cells, γ/δ T-cells, monocytes, and granulocytes in cattle (Williams et al., 1988; Stott et al., 1991; Schwartz et al., 1994; Mirsky et al., 1996; Wu et al., 1996; Panei et al., 2013), a large number of the tumor cells are derived from CD5^+^ IgM^+^ B-cell subpopulations (Schwartz and Levy, 1994). Interestingly, the full-length BLV proviral genome is maintained in each animal throughout the course of the disease (Tajima et al., 1998a). In addition, previous studies have shown that both large and small deletions of proviral genomes may be very rare events in BLV-infected cattle. Thus, the proviral loads were significantly increased at the PL stage compared with the aleukemic stage and were further increased at the lymphoma stage (Jimba et al., 2010, 2012; **Figure 2B**). These findings clearly demonstrated that the BLV proviral copy number increases with increasing severity of the disease. On the other hand, unlike BLV, HTLV-1 is associated with ATL and with the chronic neurological disorder, HAM/TSP, and induces not only a malignant tumor but also an inflammatory disease (Gessain et al., 1985; Osame et al., 1986). Although the pathogenesis of HTLV-1 is slightly different from BLV, HTLV-1, like BLV, can infect many cells in addition to T-cells, including B-cells and monocytes (Koyanagi et al., 1993; Schwartz and Levy, 1994). In contrast to BLV, defective HTLV-1 proviral genomes have been found in more than half of all examined patients with ATL (Konishi et al., 1984; Korber et al., 1991; Ohshima et al., 1991; Tsukasaki et al., 1997).

## MECHANISM OF LEUKEMOGENESIS BY BLV

Animal retroviruses, which belong to the *Alpharetrovirus* and *Gammaretrovirus* genera, induce tumors by one of two mechanisms: either by activation of the “viral oncogene” or by “insertional activation” of a cellular gene such as a proto-oncogene (Weiss et al., 1985; **Figure 1**). By contrast, BLV lacks a known oncogene (Sagata et al., 1984a,b) and does not integrate into preferred sites in their host cell genomes, which related to the disruption of the host gene but not to the suppression of viral gene expression (Murakami et al., 2011b).

Most studies of BLV-induced leukemogenesis have focused on the Tax protein because it is believed to be a potent transcriptional activator of viral gene expression. In addition to its function as a transcriptional activator, Tax induces immortalization of primary REFs (Willems et al., 1990, 1998). Furthermore, Tax cooperates with the Harvey rat sarcoma viral oncoprotein (Ha-ras) for the induction of full transformation of primary REF (Willems et al., 1990). Importantly, the Tax transformed cells induce tumors in nude mice. The ability of the Tax protein to induce immortalization may be the first step in the BLV-mediated transformation process. Moreover, after the infection of cattle and during the latency period, the expression of BLV becomes blocked at the transcriptional level (Kettmann et al., 1982; Lagarias and Radke, 1989). Such repression appears to be very important for the escape of BLV from the host’s immunosurveillance system, and later only a certain small proportion of infected animals rapidly develop a terminal disease (Gillet et al., 2007). Indeed, transcription of the BLV genome in fresh tumor cells or in fresh peripheral blood mononuclear cells (PBMCs) from infected individuals is almost undetectable by conventional techniques (Kettmann et al., 1982; Tajima et al., 2003b; Tajima and Aida, 2005). *In situ* hybridization has revealed the expression of viral RNA at low levels in many cells, and at a high level in only a few cells within PBMCs freshly isolated from BLV-infected asymptomatic animals (Lagarias and Radke, 1989). Thus, BLV infection is probably not sufficient for leukemogenesis and some additional events such as gene mutations might be involved in the leukemogenic process (**Figure 2A**). Taken together, Tax may induce immortalization of only CD5^+^ IgM^+^ B-cells among BLV-infected B-cells, CD4^+^ T-cells, CD8^+^ T-cells, γ/δ T-cells, monocytes, and granulocytes in cattle, thereby conferring a selective transformation advantage to the infected CD5^+^ IgM^+^ B-cells by a second event.

A mutation in the p53 tumor suppressor gene is one of several genetic changes known to be involved in the development of lymphoma (**Figure 2A**). The protein encoded by the p53 tumor suppressor gene plays a critical role in transducing a signal from the damaged DNA to genes that control cell cycle and apoptosis. Approximately half of the solid tumors induced by BLV in cattle (Dequiedt et al., 1995; Ishiguro et al., 1997; Zhuang et al., 1997; Tajima et al., 1998b) and three of four bovine B-cell lymphoma lines (Komori et al., 1996) were shown to harbor missense mutations in p53. By contrast, very few mutations were found in B-cells from cows with PL and none of the uninfected cattle harbored a mutated p53 gene. These observations indicate that p53 mutations frequently occur at the final stage of lymphoma in cattle. A previous study of the molecular mechanism of mutations at codons 206, 207, 241, and 242, which were identified in lymphoma, showed that these mutations may potentially alter the wild-type function of the bovine p53 protein, including the conformation and transactivator and growth suppressor activities, and then cause lymphoma (Tajima et al., 1998b). These four mutations were clearly divided into two functionally distinct groups: (i) the mutant forms with substitutions at codons 241 and 242, which were mapped within an evolutionarily conserved region and corresponded to the human “hot-spot” mutations, and had completely lost the capacity for transactivation and growth suppression while gaining transdominant repression activity in p53-null SAOS-2 cells; and (ii) the mutations at codons 206 and 207, which were located outside the evolutionarily conserved regions and partially retained the capacity for transactivation and growth suppression. Collectively, these naturally occurring mutations may potentially alter the wild-type function, and in addition, out of the four missense mutations, at least two mutations may be sufficient to cause lymphoma. However, since the other two mutations may be insufficient to induce lymphoma, it is possible that other cancer-related genes may contribute to lymphoma in concert with the p53 mutations.

A major factor involved in the clinical progression of BLV-infected animals is the bovine leukocyte antigen (BoLA; **Figure 2A**), which plays a crucial role in determining immune responsiveness (Lewin and Bernoco, 1986; Lewin et al., 1988; Zanotti et al., 1996; Takeshima and Aida, 2006). Several studies have shown that genetic variations in *BoLA-DRB3*, which is a functionally important and the most polymorphic BoLA class II locus in cattle, influence resistance and susceptibility to a wide variety of infectious diseases, including lymphoma (Aida, 2001) and PL (Xu et al., 1993; Sulimova et al., 1995; Starkenburg et al., 1997; Juliarena et al., 2008), and affect BLV proviral load (Miyasaka et al., 2013). For example, the presence of the amino acids Glu–Arg (ER) at positions 70–71 of the BoLA-DRβ chain was associated with resistance to PL in BLV-infected cattle (Xu et al., 1993). Furthermore, the *BoLA-DRB3* alleles encoding Glu, Arg, and Val at positions 74, 77, and 78, respectively, of the BoLA-DRβ chain might be associated with resistance to tumor development (Aida, 2001). In a related study, Nagaoka et al. (1999) and Konnai et al. (2003) found that the ovine leukocyte antigen (*OLA*)*-DRB1* alleles encoding the Arg–Lys (RK) and the Ser–Arg (SR) motifs at positions 70–71 of the OLA-DRβ chain are associated with resistance (RK motif) and susceptibility (SR motif) to the development of lymphoma after experimental infection of sheep with BLV. The sheep with alleles encoding the RK motif produced neutralizing antibodies against BLV and interferon-γ, eliminated BLV completely, and did not develop lymphoma (Konnai et al., 2003). The susceptibility to the monoclonal expansion of BLV-infected B-lymphocytes is thus associated with specific alleles of BoLA system.

A polymorphism in the promoter region of the tumor necrosis factor (TNF)-α gene is one of several genetic changes involved in the development of lymphoma (**Figure 2A**). A previous study found that, in sheep experimentally infected with BLV, the frequency of the TNF-α-824G allele, which has been associated with low transcription activity of the promoter/predicted enhancer region of the bovine TNF-α gene, was higher in animals with lymphoma than in asymptomatic carrier animals. In addition, a tendency was observed for increased BLV-provirus load in cattle homozygous for the TNF-α-824G/G allele compared to cattle homozygous for the TNF-α-824A/A or TNF-α-824A/G alleles. These data suggest that the observed polymorphism in the promoter region of the TNF-α gene could at least in part contribute to the progression of lymphoma in BLV infection (Konnai et al., 2006).

The BLV studies have also focused on understanding the process of signal transduction such as B-cell receptor (BCR) signaling (Alber et al., 1993), since many signal transduction factors have been implicated in leukemogenesis of B-cells in humans (Murakami et al., 2011a). For example, the immunoreceptor tyrosine-based activation (ITAM) motifs present in the transmembrane gp30 proteins of the BLV envelope are important for the incorporation of envelope proteins into the virion (Inabe et al., 1999) and are required for infectivity *in vivo* (Willems et al., 1995). In addition to the viral signaling motif, the spleen tyrosine kinase (Syk) mRNA expression was significantly increased in PL samples, whereas it was decreased in tumor samples, suggesting that Syk mRNA expression dynamics is closely related to the progression of BLV-induced disease (Murakami et al., 2011a).

## BLV Tax FUNCTION

As mentioned above, the Tax gene is a key contributor to the oncogenic potential, as well as a key protein involved in the replication of the virus. **Table 1** summarizes the functions of the Tax protein. The Tax open reading frame is mainly encoded in the pX region, and its translation occurs upstream of the *pol* stop codon. The Tax protein is modified by phosphorylation of two serine residues and is detected as a 34–38 kDa product (Chen et al., 1989; Willems et al., 1998). In addition, the Tax protein has T- and B-cell epitopes corresponding to regions 110–130/131–150 and 261–280, respectively (Sakakibara et al., 1998). One of the best characterized functions of Tax is the activation of viral transcription. The Tax protein acts on a triplicate 21 bp enhancer motif known as the Tax-responsive element (TxRE) in the U3 region of the 5′LTR, and it stimulates transactivation of the viral genome (Derse, 1987; Willems et al., 1987; Katoh et al., 1989). The TxRE consists of a cyclic AMP-response element (CRE)-like sequence, and it has been suggested that Tax binds to this element indirectly through cellular factors, such as the members of the CREB/activating transcription factor (ATF) family of basic leucine zipper proteins that have been shown to bind to the CRE-like sequence (Adam et al., 1994, 1996; Boros et al., 1995). Furthermore, the Tax protein modulates the expression of cellular genes that are involved in the regulation of cell growth (Tajima and Aida, 2002). In addition to its function in the regulation of cellular and viral transcription, the Tax protein can induce immortalization of primary REF and cooperates with Ha-Ras oncogene to fully transform the primary cells (Willems et al., 1990). On the other hand, the transactivation and transformation of Tax may be independently induced by each mechanism, since phosphorylation of Tax is required for its transformation but not for its activation (Willems et al., 1998). Moreover, the expression of Tax in primary ovine B-cells, which depends on CD154 and interleukin-4, affects B-cell proliferation, cell cycle phase distribution, and survival, leading to cytokine-independent growth (Szynal et al., 2003). This immortalization process is also associated with increased B cell leukemia/lymphoma 2 (Bcl-2) protein levels, nuclear factor kappa B (NF-κB) accumulation, and a series of intracellular pathways that remain to be characterized (Klener et al., 2006). In addition, Tax inhibits base-excision DNA repair of oxidative damage, thereby potentially increasing the accumulation of ambient mutations in cellular DNA (Philpott and Buehring, 1999).

## NEGATIVE REGULATION OF BLV Tax BY THE REGION BETWEEN RESIDUES 240–265

**FIGURE 3:**
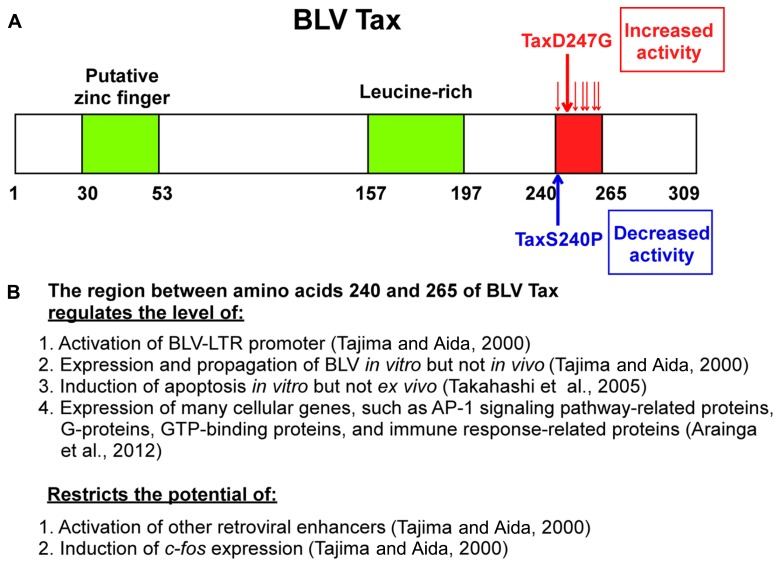
**Schematic representations of BLV Tax protein mutants and function.** **(A)** Missense mutations between amino acids 240 and 265 containing D247G and S240P influence the transactivation activity of the BLV Tax protein. A putative zinc finger structure (amino acids 30–53) and a leucine-rich activation domain (amino acids 157–197) are also indicated. **(B)** Multiple functions of the region between amino acids 240 and 265.

Our studies (Tajima and Aida, 2000) demonstrated new functions of the region between amino acids 240 and 265 of BLV Tax. As shown in **Figure 3**, a series of mutants with at least one amino acid substitution between amino acids 240 and 265 of BLV Tax were identified, including TaxD247G and TaxS240P, which exhibit an enhanced ability to stimulate and reduce viral LTR-directed transcription respectively, compared to the wild-type protein (Tajima and Aida, 2000). Transient expression analysis revealed that the TaxD247G mutant increased the production of viral protein and particles from a defective recombinant proviral BLV clone to a greater extent than the wild-type Tax (TaxWT). Conversely, the TaxS240P mutant was unable to induce the release of viral particles. The microarray data in human HeLa cells and its validation of differentially expressed genes at the RNA and protein levels in bovine 23CLN cells revealed several alterations in genes involved in many cellular functions such as transcription, signal transduction, cell growth, apoptosis, and the immune response (Arainga et al., 2012). In both of human HeLa cells and bovine 23CLN cells, the TaxD247G mutant induced higher gene expression compared with TaxWT and TaxS240P and many of these genes were expressed at the lowest level in the TaxS240P-transfected cells. In particular, our results showed that Tax activates the proteins which are involved in activator protein 1 (AP-1) signaling pathway [FBJ osteosarcoma oncogene (FOS), jun proto-oncogene (JUN), etc.] via interactions with other transcriptional pathways (G-protein, GTP-binding proteins, etc.). Likewise, the TaxD247G mutant induced apoptosis in transfected cells more effectively than the TaxWT (Takahashi et al., 2005). These results suggest that the region between amino acids 240 and 265 of the Tax protein might act as a negative regulatory domain, and missense mutations in this region might lead to enhanced transactivation activity of Tax, expression of many cellular genes and induction of apoptosis. Our results raise the possibility that the target sequence specificity of retroviral enhancers of Tax might be limited by this region because TaxD247G, but not TaxS240P, was found to activate other retroviral enhancers such as HTLV-1, HIV-1, and mouse mammary tumor virus (MMTV) and Moloney murine leukemia virus (M-MuLV), and *c-fos*, which are not activated by TaxWT (Tajima and Aida, 2000; **Figure 3B**). The microarray data also raised the possibility that BLV Tax regulates the innate immune response (**Figure 3B**): the largest group of downregulated genes was related to the immune response, and the majority of these genes belonged to the interferon family of antiviral factors, such as interferon-induced protein with tetratricopeptide repeats 1 (IFIT1; Arainga et al., 2012). Interferons are major components of the innate immune system, and are recognized for their antiviral function in addition to their antiproliferative and immunomodulatory effects on cells (Hu et al., 1993). It is likely that BLV Tax downregulates the innate immune response, thereby increasing the production of viral protein.

**FIGURE 4:**
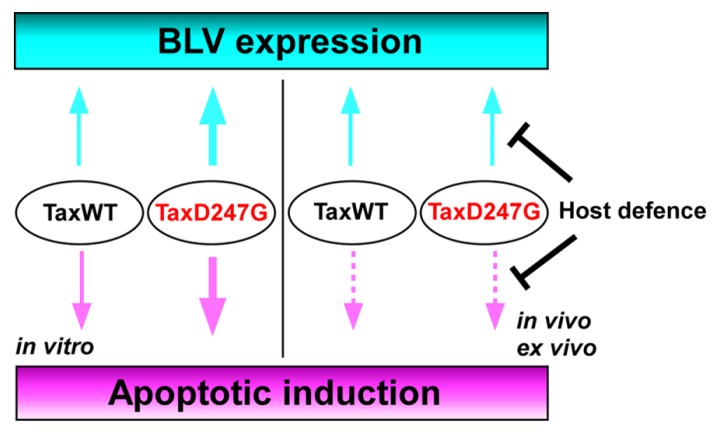
**Proposed mechanism for the regulation of BLV expression and apoptosis induction in TaxD247G-encoded BLV-infected sheep**.

An infectious molecular clone of BLV encoding the TaxD247G was examined for the viral expression and propagation, as well as for the induction of apoptosis in a sheep model** (Tajima et al., 2003a; Takahashi et al., 2004, 2005). Interestingly, the infectious molecular clone of BLV encoding the TaxD247G produced more viral particles and was transmitted at an elevated rate *in vitro*, but with no significant differences in the proviral load and the expression of viral RNA between sheep experimentally injected with BLVs encoding the TaxWT or the mutant TaxD247G proteins (Tajima et al., 2003a). These findings suggest the presence of a dominant host defense mechanism regulating BLV–LTR-directed transcription by Tax that may play an important role in viral silencing *in vivo* (**Figure 4**). Likewise, although the transient expression of TaxD247G induced apoptosis** in** transfected cells *in vitro* more effectively than TaxWT, higher level of protection against apoptosis was observed in PBMCs isolated from sheep infected with TaxD247G-encoded BLV compared to TaxWT-encoded BLV (Takahashi et al., 2005; **Figure 4**). These findings demonstrate that TaxD247G has an increased potential to induce apoptosis, which could be beneficial for BLV propagation like other viruses (Wurzer et al., 2003; Richard and Tulasne, 2012). One possible explanation for our results might be that TaxD247G-induced apoptosis is modulated by a dominant mechanism *ex vivo*, so the function might be suppressed.

## CONCLUSION

BLV is the etiologic agent of EBL, which is the most common neoplastic disease in cattle. It infects cattle worldwide, thereby imposing a severe economic burden on the dairy cattle industry. In this review, we evaluated existing information on the mechanism of BLV-induced leukemogenesis. We propose that, since BLV Tax induces immortalization of only CD5^+^ IgM^+^ B-cells within BLV-infected B-cells, CD4^+^ T-cells, CD8^+^ T-cells, γ/δ T-cells, monocytes, and granulocytes in cattle, it may confer a selective transformation advantage to the infected CD5^+^ IgM^+^ B-cells by a second event, such as p53 mutation, polymorphisms of BoLA, or the promoter region of the TNF-α gene. We also propose new functions of the region between amino acids 240 and 265 of BLV Tax (**Figure 3**). Namely, the transactivation activity and target sequence specificity of BLV Tax might be limited or negatively regulated by this region. The most interesting point regarding the ability of TaxD247G to enhance BLV expression and apoptotic induction *in vitro* is that it might be suppressed *in vivo* or *ex vivo*. Thus, we hypothesize that there could be dominant mechanisms controlling the functions of TaxD247G *ex vivo* and *in vivo*, as shown in **Figure 4**. For HTLV-1, it has been reported that CD8^+^ cell-mediated cytotoxic T-lymphocytes (CTLs) target Tax-expressing cells, thereby reducing the number of infected cells (Hanon et al., 2000). Likewise, BLV-infected cells expressing Tax may be exposed to the host defense system, and BLV may evolve in a manner that promotes the shielding of their potential abilities. Therefore, a strong transactivation activity of BLV Tax might not be advantageous for the propagation of BLV *in vivo*. Taken together, the findings discussed in this review suggest that there might be a dominant mechanism involved in the induction of apoptosis and expression of HTLV-1 *in vivo*. To address our hypothesis, it seems necessary to evaluate whether possible host responses against BLV infection, such as the induction of CTLs, genetic, and epigenetic alterations in apoptosis-regulatory genes, and DNA and chromatin modifications of BLV promoter for the suppression of viral expression, could be enhanced in animals infected with TaxD247G-encoded BLV. Thus, future investigations of the relationship between apoptosis and viral expression using BLV containing the mutant D247G Tax as a model will broaden our understanding of the replication and propagation of HTLV-1, and leukemia progression.

## Conflict of Interest Statement

The authors declare that the research was conducted in the absence of any commercial or financial relationships that could be construed as a potential conflict of interest.
